# Isolation, Characterization and Structure Elucidation of a Novel Lantibiotic From *Paenibacillus* sp.

**DOI:** 10.3389/fmicb.2020.598789

**Published:** 2020-11-24

**Authors:** Jerzy Karczewski, Stephen P. Krasucki, Papa Nii Asare-Okai, Carl Diehl, Andrew Friedman, Christine M. Brown, Yukari Maezato, Stephen J. Streatfield

**Affiliations:** ^1^Fraunhofer USA Center for Molecular Biotechnology, Newark, DE, United States; ^2^Department of Chemistry and Biochemistry, University of Delaware, Newark, DE, United States; ^3^SARomics Biostructures AB, Lund, Sweden

**Keywords:** antimicrobial peptide, lantibiotic, NMR, stability, antibiotic resistance, MRSA

## Abstract

We have isolated and characterized a novel antibacterial peptide, CMB001, following an extensive screening effort of bacterial species isolated from diverse environmental sources. The bacterium that produces CMB001 is characterized as a Gram (+) bacillus sharing approximately 98.9% 16S rRNA sequence homology with its closest match, *Paenibacillus kyungheensis*. The molecule has been purified to homogeneity from its cell-free supernatant by a three-step preparative chromatography process. Based on its primary structure, CMB001 shares 81% identity with subtilin and 62% with nisin. CMB001 is active mainly against Gram-positive bacteria and Mycobacteriaceae but it is also active against certain Gram-negative bacteria, including multi-drug resistant *Acinetobacter baumannii*. It retains full antibacterial activity at neutral pH and displays a low propensity to select for resistance among targeted bacteria. Based on NMR and mass spectrometry, CMB001 forms a unique 3D-structure comprising of a compact backbone with one α-helix and two pseudo-α-helical regions. Screening the structure against the Protein Data Bank (PDB) revealed a partial match with nisin-lipid II (1WCO), but none of the lantibiotics with known structures showed significant structural similarity. Due to its unique structure, resistance profile, relatively broad spectrum and stability under physiological conditions, CMB001 is a promising drug candidate for evaluation in animal models of bacterial infection.

## Introduction

For many decades, natural products were one of the major sources of new compounds or templates for discovery of novel drugs. In particular, the majority of currently prescribed antibiotics are derived from natural sources. Based on a recent analysis, 78 antibiotics, or just over 48% of all approved antibiotics, are either natural products or were derived from them (Newman and Cragg, 2020). Antimicrobial peptides are produced by a variety of organisms as a first line of defense against competing or infecting pathogens. The benefits, challenges, and opportunities presented by antimicrobial peptides as drug candidates have been reviewed recently (Magana et al., 2020). Lantibiotics are naturally occurring, ribosomally synthesized cationic antimicrobial peptides produced by Gram-positive bacteria. These polycyclic peptides contain several post-translationally modified amino acids: dehydroalanines, dehydrobutyrines and lanthionines (McAuliffe et al., 2001). Lantibiotics are classified into several separate classes (types) based on their structural arrangement. The most studied class, type A(I) includes multiple variants of nisin (A-Z), subtilin, entianin and ericin (McAuliffe et al., 2001). The prototype lantibiotic, nisin, was first described as “a relatively insoluble substance that inhibited growth of certain micro-organisms” (Gowans et al., 1952). Nisin has been widely used in the food industry as a preservative to inhibit growth of pathogens of concern for dairy foods, specifically *Listeria monocytogenes* and *Staphylococcus aureus* (Ibarra-Sanchez et al., 2020) as well as to extend shelf life of meat, juices and other food products (Juneja et al., 2012). Due to its successful use in the food industry, nisin and its analogs have also been considered for pharmaceutical applications (Shin et al., 2016). Recent studies using high resolution NMR spectroscopy revealed the molecular basis for the formation of nisin:lipidII complexes (Hsu et al., 2004) and the topology of the complex within a membrane pore (Medeiros-Silva et al., 2018). Unlike other lipid II-binding antibiotics, such as vancomycin, which binds to the C-terminal d-Ala-d-Ala of the pentapeptide (Ng and Chan, 2016), nisin and likely other lantibiotics wrap around the pyrophosphate linkage of lipid II (Hsu et al., 2004). Since the pyrophosphate and lipid parts of lipid II cannot be directly modified by gene mutations and since some lantibiotics form pores within the bacterial membrane, the emergence of resistance against lantibiotics is likely more difficult to develop than against vancomycin and related antibiotics (Draper et al., 2015).

Major hurdles preventing the development of nisin for treatment of human infections were related to its limited stability (Rollema et al., 1995) and low solubility at physiological pH (Liu and Hansen, 1990) and at physiological NaCl concentrations (Rollema et al., 1995). In addition, chemical inactivation of lanthionines by oxidation (Wilson-Stanford et al., 2009) and *in vivo* toxicity (Gowans et al., 1952) have been reported. Multiple naturally occurring nisin variants have been discovered and studied, including nisin F (De Kwaadsteniet et al., 2009), nisin P (Garcia-Gutierrez et al., 2020), nisin V (Campion et al., 2013), and nisin H (O’Connor et al., 2015). In addition, other nisin-like lantibiotics sharing a similar biosynthetic pathway and assignment of lanthionine bridges have been discovered, including subtilin, entianin, and ericin (Dischinger et al., 2014). Despite structural similarities there is considerable variation in physicochemical properties, antibacterial activity and selectivity among lantibiotics (Dischinger et al., 2014; Simons et al., 2020). Mutagenesis studies were undertaken to generate variants of lantibiotics with improved stability (Liu and Hansen, 1992; Rollema et al., 1995), altered antimicrobial activity (Field et al., 2008, 2012; Li et al., 2018) or improved resistance profiles (Lagedroste et al., 2019; Zaschke-Kriesche et al., 2019a,b). Such mutagenesis studies, combined with NMR structural analysis, helped to identify specific hot-spots important for activity of nisin (Medeiros-Silva et al., 2018).

In this study, we describe the purification, NMR structure determination, and biological activity of a novel lantibiotic isolated from *Paenibacillus sp*. We demonstrate that this compound, which we designate CMB001, is active against a wide range of Gram-positive drug-resistant bacteria and selected Gram-negative bacteria, is stable in physiological solutions and is less prone to select for resistance compared to nisin. The structural analysis reveals the compact structure of CMB001, distinct from nisin and other lantibiotics. It is anticipated that due to its stability and solubility, the *in vivo* efficacy of CMB001 will be greatly improved over those of nisin and other lantibiotics.

## Materials and Methods

### Materials

Chemical grade reagents were obtained from Sigma-Aldrich and Fisher Chemicals. Microbiological media was obtained from Becton, Dickinson, and Company (BD).

### Indicator Strains

Common bacterial strains were obtained from American Type Culture Collection (ATCC). Drug-resistant and drug-susceptible *S. aureus* and *A. baumannii* stains (CH11 and CH-36-53) were obtained from Christiana Hospital (Wilmington, DE, United States). *Bacillus anthracis* and *Yersinia pestis* were in the collection of the United States Army Medical Research Institute of Infectious Disease (Fort Detrick, MD, United States).

### Fermentation of the Producer Strain

The producer strain was isolated from Fraunhofer United States’s bacterial library by sequential plating and purification of single colonies. It was identified by 16S rRNA sequencing (GENEWIZ). Antimicrobial activity screening was done by a commonly used “spot-on-lawn” assay (Vijayakumar and Muriana, 2015). The test was performed by preparing a 3 mL of soft-agar (0.8%) containing 10 μL of freshly grown *S. aureus* ATCC 29213 strain (at OD600 = 0.5). Soft-agar cell mixture was poured on the plate and left for several minutes to solidify. Antimicrobial activity was evaluated by spotting 10 μL of CMB001 cell-free liquid culture supernatant onto the plate. The zone of inhibition (ZOI), indicating activity, was observed after overnight incubation at 32°C. To generate material for purification, bacteria were grown in shaker flasks or in a 10 L bioreactor (Sartorius BIOSTAT B plus). Seed cultures were inoculated BD Difco Terrific Broth (TB) medium and were incubated at 32°C overnight. Growth media (TB + 1.25% starch) was prepared in the fermenter vessel, autoclaved and cooled overnight. Seed cultures were inoculated into the bioreactor at 5% (v/v), aseptically. After inoculation, fermentation was conducted at 32°C. The presence of antibacterial activity in the media was monitored throughout fermentation, with peak activity typically reached after 24-27 h. At around 24 h, cultures were harvested from the bioreactor and clarified by centrifugation (6000 × *g* for 15 min). The supernatant was filtered through a 0.2 μm PES filter (Thermo Fisher Scientific). Antimicrobial activity was confirmed by a lawn-spot test against *S. aureus* grown in BD Bacto Tryptic soy broth (TSB) overnight at 37°C. The filtered supernatant was stored at 14°C until further processing.

### Purification of CMB001

An AKTA Pure 150 system was used to purify the antimicrobial active component from supernatants recovered from fermentations. Filtered supernatant was brought to 0.7 M ammonium sulfate, supplemented with 20 mM 2-(N-morpholino)ethanesulfonic acid (MES) and pH was adjusted to 6.0, prior to loading onto a 200 mL GE Phenyl Sepharose HP column equilibrated with 20 mM MES, pH 6.0 containing 0.7 M ammonium sulfate. The column was washed and then step eluted with 0.3 M ammonium sulfate, 20 mM MES pH 6. Active fractions were pooled and diluted to a conductivity of 7 mS/cm. The sample was loaded onto a 200 mL GE SPHP column and eluted at 30% 2 M NaCl, 20 mM MES pH 6. Active fractions were pooled and brought to 5% acetonitrile (ACN), 0.1% trifluoroacetic acid (TFA). The sample was loaded onto a GE Source 30 RPC column and eluted from 5-95% ACN, 0.1% TFA for 20 min. Active fractions were pooled and concentrated on a freeze-drier (FreeZone, Labconco) and further purified on a XSelect CSH preparative C18 column (5 μm, 19 × 250 mm, Waters Corp.) using a Waters HPLC chromatography system equipped with a QDa quadrupole mass spectrometer (LC/MS). The following MS settings were used: cone voltage − 20 V, capillary voltage − 0.8 kV, probe temperature − 600°C. Active fractions were concentrated on a freeze-drier, dissolved in distilled water at 5-25 mg/mL and stored frozen at −20°C. Purified product was quantified by analytical HPLC (Agilent Eclipse XDB-C18 5 μm 4.6 × 150 mm) and analyzed by high resolution mass spectroscopy (Q Exactive orbitrap, Thermo Scientific). Nisin was purified from a commercial 2.5% solution (Sigma-Aldrich) using the procedure described above for CMB001. Polymyxin B nonapeptide (PMBN) was prepared by papain treatment of polymyxin B as previously described (Vaara, 1988).

To test for solubility, CMB001 and nisin were incubated in 50 mM sodium citrate, pH 5.0 or in 50 mM phosphate, pH 7.0 at 37°C for 0-24 h and then centrifuged at 16,000 × *g* for 15 min. Peptide content in the supernatant was then measured using analytical HPLC.

### Antimicrobial Susceptibility Testing

The minimum concentration required to inhibit 90% of bacterial growth (MIC) was determined by broth microplate dilution. General guidelines from the Clinical and Laboratory Standards Institute (CLSI) were followed with minor modifications of incubation conditions needed for specific indicator strains. Briefly, indicator organisms were prepared to 0.5 McFarland standard and diluted in broth (Mueller-Hinton Broth or Tryptic Soy Broth) to yield 50,000 CFU/well and deposited in a 96-well microplate. Drugs to be tested were prepared in sterile water to 10× the final concentration and 10 μL was combined with 90 μL of indicator. Microplates were incubated at 37°C overnight or until turbid for slower growing organisms. Efficacy of the drug was evaluated by absorbance at 600 nm determined with a BioTek Cytation5 imaging reader (Biotek).

### Frequency of Resistance (FoR)

Mueller-Hinton agar (MHA, BD Difco: BD 225250) was prepared according to the manufacturer’s instructions, autoclaved and cooled to 60°C in a water bath. Appropriate amounts of drugs to be tested (1× to 10× MIC) were added to cooled MHA, mixed thoroughly, poured into sterile petri dishes and allowed to cool overnight at ambient temperature. Indicator bacterial cultures were streaked onto MHA plates and incubated at 37°C overnight. These plates provided inoculum to start 20 mL liquid cultures at an OD_600_ of 0.05. Bacterial cultures were incubated at 37°C on a shaker incubator and grown to late log phase (OD_600_ of approximately 1.5), washed with 1× phosphate-buffered saline (PBS) and plated onto MHA plates containing test drugs at multiple MICs. Plates were incubated overnight at 37°C. The frequency of resistance was calculated by dividing the number of resistant colonies recovered on the CMB001 containing plate by the total CFU in the initial inoculum, corresponding to the viable cell count.

### Anti-biofilm Activity

Anti-biofilm activity of CMB001 was evaluated using the method of Cruz et al. (2018) with minor modifications. Briefly, aliquots of *S. aureus* ATCC 29213 suspended in TSB supplemented with 2.5 g/L glucose were incubated in Nunclon polystyrene 96-well plates (Thermo Fisher Scientific), for 24 h, after which the wells were washed to remove planktonic cells. Fresh media was added, and the plates were further incubated overnight at 32°C to allow for biofilm formation. CMB001 was added to the final concentration 1.6-100 μg/mL and incubated with the biofilm for 4 h. To assess the viability of cells in biofilm plates were washed and stained with the cell permeable resazurin-based PrestoBlue Cell Viability Reagent (Thermo Fisher Scientific) and fluorescence measured using SpectraMax M microplate reader (Molecular Devices). To test in a “pre-treatment” format, the surfaces of 96-well plates were coated with CMB001, then washed to remove unbound peptide. *S. aureus* ATCC 29213 suspended at an OD_600*nm*_ of 1 was added to peptide-coated plates and incubated for 1 or 24 h at 37°C. At the end of the incubation period, non-adherent cells were removed, wells were washed 3× in PBS and cells were stained with PrestoBlue, as described above. Percent cell viability was calculated based on fluorescence after treatment relative to control fluorescence, measured in the presence of media.

### Growth Curve Analysis

*Staphylococcus aureus* ATCC 29213 and multi-drug resistant (MDR) CH-11 cultures were grown in 96-well plates with constant shaking. OD_600*nm*_ was measured every 30 min using a Spectra Max microplate reader (Molecular Devices). To assess its effect on target cultures, CMB001, or vehicle control, was added at mid-log phase at 2× its minimum inhibitory concentration (MIC). Cell viability was determined by CFU/mL counts by sampling the cells pre- and post-treatment with CMB001, by plating on agar, incubating at 37°C, and counting CFUs.

### Membrane Depolarization Assay

*Staphylococcus aureus* (ATCC 29213) cells were cultured overnight in TSB, then washed once and resuspended at an OD_600*nm*_ of 0.1 in assay buffer containing 5 mM HEPES, 5 mM glucose and 0.1 M KCl, at pH 7.4. Bacteria were dispensed into a 96-well black plate and 3,3′-dipropylthiadicarbocyanine iodide [diSC3(5)] (Sigma-Aldrich Co., LLC, MO, United States) was added to a final concentration of 1 μg/mL and incubated with the cells for 30 min. Test peptide was then added to the final concentration 1-50 μg/mL, the mixture incubated for 30 min and fluorescence measured using a Cytation 5 microplate reader (BioTek) with an excitation wavelength of 622 nm and emission at 670 nm.

### Amino Acid Composition

An 800 μg quantity of the peptide was reduced in 20 mM EDTA, 200 mM Tris-HCl pH 8, 1.2 M guanidine-HCl and 15.9 mg Raney nickel. The sample was incubated at 55°C for 36 h. The Raney nickel was removed by 3 rounds of centrifugation, followed by desalting with a peptide desalting spin column (Thermo Fisher Scientific). The sample was lyophilized and re-dissolved in water. The peptide was then digested using a rapid hydrolysis method (Tsugita and Scheffler, 1982) in the presence of HCl and TFA at 135°C for 3 h. Composition analysis of reduced and unreduced hydrolyzed samples was performed on an analytical LC/MS equipped with a Q Exactive orbitrap (Thermo Fisher Scientific, Waltham, MA, United States) and Intrada 50 × 3 mm amino acid column (Imtakt, Portland, OR, United States).

### NMR Structure Determination

A sample of CMB001 was prepared for NMR studies by dissolving 9.5 mg of peptide in 500 μl DMSO-d6 to a final concentration of 5.7 mM. NMR spectra were collected at 700 MHz on a 700 MHz Bruker Avance III HD magnet equipped with a 5 mM QCI cryoprobe at 298 K. 1H-1H TOCSY (40 and 80 ms mixing time), 1H-1H NOESY (100 and 300 ms mixing time), 1H-1H DQF-COSY, 1H-15N HSQC, 1H-13C HSQC and 1H-13C HMBC spectra were acquired. Spectra were processed using the NMRPipe software suite (Delaglio et al., 1995). The processing scheme used for all spectra was solvent suppression in direct dimension, squared cosine apodization, phasing and zero filling in both dimensions and polynomial baseline correction in direct dimension. Spectra were referenced in the direct dimension using the DMSO-d6 reference signal. NMR spectra were assigned using the CCPNMR software suite (Vranken et al., 2005).

Distance restraints from NOEs were generated using the CCPNMR software suite (Vranken et al., 2005). Dihedral angle restraints were generated using the TALOS-N program using assigned chemical shifts. Structure calculations were performed using the GROMACS software suite. Structure calculations were initialized from an extended structure generated by in-house software. Lanthionine bridges were generated by setting the residues in the lanthionine bridge to Ser/Thr and Cys and then applying distance restraints and the inbuilt functions in GROMACS to generate new covalent bonds. The parameters for DHA and MDH were taken from the SwissSideChain homepage (Gfeller et al., 2013). After generation of the lanthionine bridges, the extended structure was energy minimized, solvated in DMSO and equilibrated. Structure determination was performed in an iterative fashion until achieving a converged structure ensemble of 15 structures. Structure quality was determined using in-house software (Saromics) and Molprobity (Chen et al., 2010).

## Results

### Discovery, Isolation, and Solubility of CMB001

**FIGURE 1:**
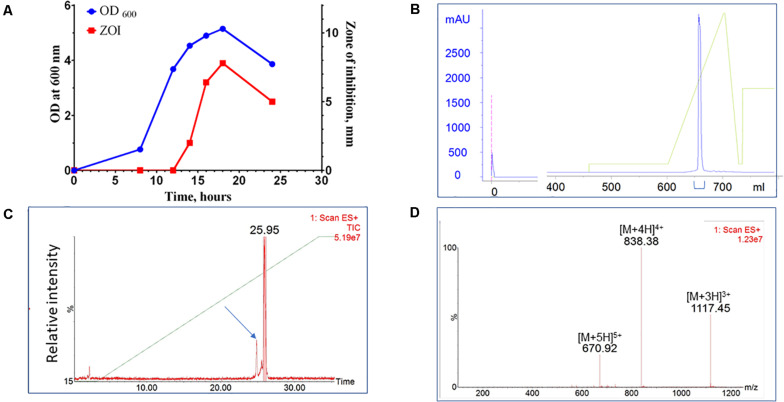
Purification of CMB001. **(A)** Growth curve of *Paenibacillus sp.* (OD600) of and secretion of antimicrobial activity (ZOI). **(B)** Preparative reverse-phase chromatography of CMB001. UV detection at 215 nm. **(C)** Total mass chromatogram (TIC) by ACQUITY QDa. The arrow indicates the minor impurity separated from the main product. **(D)** ES + spectrum of the major peak (eluted at 25.95 min).

A bacterial library assembled from environmental, predominantly soil, samples was screened for species with antimicrobial activity. Approximately 4,500 colonies were screened for antimicrobial activity against *S. aureus*. Approximately 5% exhibited antimicrobial activity and 10% of those strains (0.5% of all strains) were identified as unique by 16S rRNA sequencing analysis. One such novel isolate was identified with activity against a range of Gram-positive bacterial species. This new isolate is a rod-shaped bacterium and, based on 16S ribosomal RNA sequencing, shares 98.9% similarity with *Paenibacillus kyungheensis*. It therefore constitutes a new species or highly diverged strain. Multiple parameters of fermentation were screened, including temperature, time, and composition of growth medium. The optimal secretion of antimicrobial activity was achieved with 1.25% starch as the carbon source. The activity was detected in supernatant after 12 h of fermentation and reached its maximum after 24-27 h (Figure 1A). To provide material for purification, the producer bacteria was grown in shaker flasks (at 3-6 L scale) or in a bioreactor (at 6 to 10 L scale). Following ultrafiltration to remove cell debris, the active compound was captured and concentrated using hydrophobic interaction chromatography (Phenyl-Sepharose). The material was further fractionated using cation exchange chromatography (SP-HP) and submitted to large-scale reversed-phase chromatography (Figure 1B). Based on analytical UPLC, the typical purity of the product obtained using this three-step process was equal to or greater than 95%. The material was further fractionated using preparative HPLC equipped with an ACQUITY QDa detector (Waters Corp). The major advantage of this additional step was to separate components based on specific mass, so that minor impurities (Figure 1C, arrow), could be eliminated and the purity of the target increased to >99% (Figure 1D).

**FIGURE 2:**
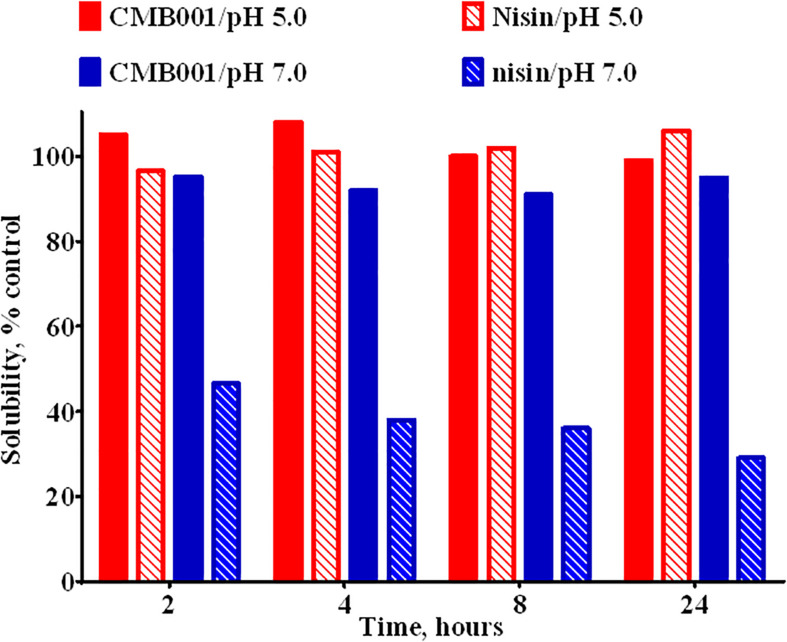
Solubility of CMB001 and nisin in aqueous buffers. Solubility of 1 mg/mL solutions of CMB001 and nisin at pH 5 and 7. Peptide solutions were incubated at the indicated pH values for 2-24 h, centrifuged and peptide content in the supernatant measured by HPLC. Solubility is expressed as% of that for the initial solution.

Due to interference form media components we were unable to accurately estimate the concentration of the active component in the original culture. Based on comparative analysis of zones of inhibition produced by unprocessed culture against media spiked with known amounts of purified peptide, the estimated concentration at the time of harvest was ∼20-40 μg/L. Typical process yields from an 8 L fermentation are 50-100 mg of purified product (6-12 mg/L, 30% process recovery). Based on amino acid composition analysis and mass spectrometry, the final product is a peptide with molecular weight of 3,346.6 Da (Figure 1D and Supplementary Figure 1) that we have designated CMB001. The maximum solubility of CMB001 in water has not been established, but it is typically diluted to 15-25 mg/mL and stored at −70°C for up to a year without significant loss of activity. Depending on the final application, CMB001 can be formulated in water, PBS or saline. To compare solubility, 1 mg/mL solutions of CMB001 and nisin were formulated in sodium phosphate pH 7.0 or sodium acetate, pH 5.0 and incubated at 37°C. At pH 5.0, both peptides remained in a solution for up to 24 h. In contrast, at pH 7.0, while CMB001 remained fully soluble for at least 24 h (Figure 2), nisin formed a visible precipitate, resulting in a significantly lower concentration in the soluble fraction, reducing to approximately 45% immediately after pH adjustment and to 30% of the starting concentration after 24 h. Similar solubility was observed in phosphate buffered saline (PBS, data not shown).

### Antimicrobial Spectrum of CMB001

The antimicrobial activity of CMB001 was assessed against a range of bacterial pathogens (Table 1). Purified CMB001 inhibited all Gram-positive bacteria against which it was tested, including *S. aureus* (ATCC 29213,CH-11 and USA300, MIC = 2-4 μg/mL), *Enterococcus faecium*, *Enterococcus faecalis, Clostriduim difficile*, and *B. anthracis*, including antibiotic resistant strains (MIC = 2-4 μg/mL). In parallel, we confirmed the activity of nisin against selected isolates, including *S. aureus* ATCC 29213 (MIC = 4-8 μg/mL), MDR *S. aureus* CH-11 (MIC = 8 μg/mL) and *S. aureus* USA300 (MIC = 4 μg/mL), Table 1. CMB001 also inhibited select Gram-negative pathogens, including drug-resistant *A. baumannii* CH-46 (MIC = 10μg/mL) (Table 1) as well as a panel of clinical strains representative for *Acinetobacter sp*. (Supplementary Table 1), *Escherichia coli* DC0 and DC2 (MIC = 62 μg/mL),*Yersinia pestis* (MIC = 16 μg/mL). By comparison, tested against select Gram-negative isolates, nisin inhibited growth of *A. baumannii* CH-46 (MIC = 10 μg/mL) but was inactive against for *E. coli* DC0 and DC2 and *K. pneumoniae* (MIC > 200 μg/mL). While most of the class I lantibiotics share a common mode of action against Gram-positive bacteria, their mode of action against Gram-negative bacteria is not fully understood. The DC2 outer membrane deficient mutant of *E. coli* was equally sensitive as DC0 to CMB001, indicating action on the outer membrane may not be important for activity of CMB001. By contrast, the DC2 mutant has been shown to be more sensitive to various other antibiotics, including erythromycin, novobiocin and rifampin (Clark, 1984). In addition, pre-treatment of *A. baumannii* ATCC19606 with the polymyxin B nonapeptide PMBN at up to 15 μg/mL did not potentiate activity of CMB001 against this strain (not shown). PMBN is a derivative of polymyxin B lacking direct antibacterial activity but capable of increasing permeability of the outer membrane and sensitizing Gram-negative bacteria to other antibiotics (Vaara, 2019). Thus, it is unlikely that the action of CMB001 toward susceptible Gram-negative bacteria is constrained by the outer membrane.

**TABLE 1:** Antimicrobial activities of CMB001.

Bacteria	Strain	MIC (μg/mL)	Nisin (μ g/mL)
*S. aureus*	ATCC 29213	2-4	4-8
MDR *S. aureus*	CH-11	4	8
Methicillin-resistant *S. aureus* (MRSA)	USA300	4	4
*E. faecalis*		3.4	n/d
Vancomycin-resistant *E. faecium*	NRBC 100486	4	4-8^*c*^
*Bacillus anthracis*	Ames	8^*a*^	n/d
*Bacillus anthracis*	Sterne	4^*a*^	n/d
*Clostridium difficile*	VPI 10463	2	3.2^*d*^
*M. tuberculosis*	Mtb H37Rv	0.3^*b*^	n/d
*M. smegmatis*	ATCC 14468	4	n/d
Multi-drug resistant *A. baumannii*	CH-46	10	10
Multi-drug resistant *A. baumannii*	ATCC 19606	15	n/d
Multi-drug resistant *P. aeruginosa*	ATCC 27853	125	n/d
*K. pneumoniae*	ATCC 33495	125	n/d
*E. coli* DC0	DC0	62.5	> 200
*E.coli* DC2	DC2	62.5	> 200
*Yersinia pestis*	CO92	16^*a*^	n/d

*^*a*^Rekha Panchal, USAMRIID, personal communication. ^*b*^Magdalena Klink, PAS, Poland, personal communication. ^*c*^Piper et al. (2009), ^*d*^Lay et al. (2016).*

### Characterization of CMB001 Action Against *S. aureus*

**FIGURE 3:**
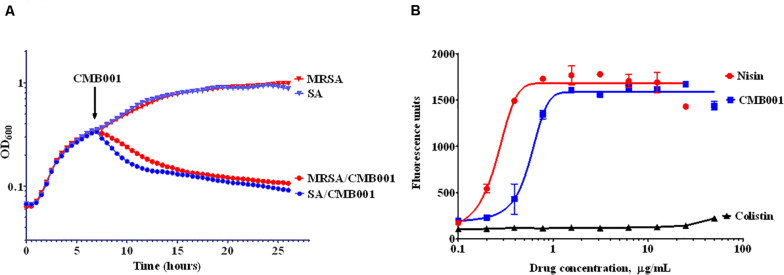
Mechanism of inhibitory action. Growth curves of *S. aureus* ATCC 29213 (SA) and Methicillin-resistant *S. aureus* (MRSA) in the presence or absence of CMB001 **(A)**, and membrane depolarization of *S. aureus* in response to CMB001, nisin or colistin **(B)**.

The turbidimetric method was used to monitor the growth rate of *S. aureus* (Figure 3A). Control cells were treated with vehicle and reached stationary phase after approximately 15 h. Similar OD trends over time were observed for the MRSA strain. The addition of 4 μg/mL of CMB001 during mid-exponential growth resulted in a rapid arrest of growth followed by a gradual decrease in turbidity for both stains, indicating cell lysis. Furthermore, the viable counts of *S. aureus* (CFU/mL) was dramatically reduced from 1 × 10^8^.^5^ to 1 × 10^4^ after 1 h of spiking with CMB001, and finally reached 0 at 7 h post-CMB treatment.

To measure the ability of CMB001 to depolarize bacterial cell membranes, we used the cationic voltage-sensitive dye, DiSC3(5), which is normally translocated into the lipid bilayer where it self-quenches. After the cell membrane is damaged, the dye is released into the medium, increasing fluorescence. Treatment with CMB001 or nisin resulted in a similar, rapid and dose-dependent increases in fluorescence for *S. aureus* ATCC 29213, indicating that integrity of the cell membrane was compromised (Figure 3B). Fluorescence reached its maximum intensity at 3 μg/mL for CMB001 and at 1.5 μg/mL for nisin.

**FIGURE 4:**
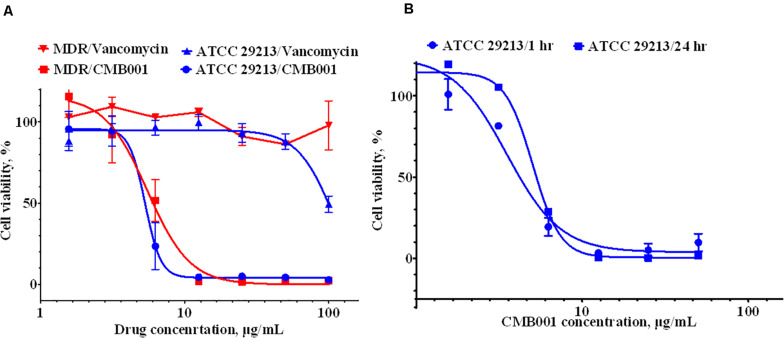
Anti-biofilm activity of CMB001. **(A)** Viability of pre-formed biofilms when treated with CMB001 or vancomycin *S. aureus* ATCC 29213 (SA) and MDR strain CH-11. **(B)** Inhibition of bacterial (*S. aureus* ATCC 29213) adhesion to a surface at 1- and 24-h following pre-coating with CMB001.

The anti-biofilm activity of CMB001 against *S. aureus* strains was evaluated under static conditions. The viability of cells, measured using the resazurin-based Live/Dead cell assay exposed to CMB001 decreased in a dose-dependent manner, with a 50% reduction of cell viability (EC50) achieved at ∼ 6 μg/mL for both *S. aureus* ATCC 29213 (SA) and methicillin-resistant *S. aureus*, MDR strain CH-11 (Figure 4A). In contrast, treatment of these biofilms with vancomycin was partially effective only against SA and only at the highest concentration 100 μg/mL. Since CMB001 reduced the cell viability of pre-formed biofilms, this suggested that the compound was able to penetrate the biofilm matrix and act directly on cells within the biofilm (but likely not directly disrupt the biofilm matrix).

To test whether CMB001 can prevent formation of biofilms, the wells of polystyrene microtiter plates were pretreated with CMB001 and then washed to remove unbound peptide. *S. aureus* ATCC 29213 was then added to the wells and incubated for 1 to 24 h. In this case, the dose-response to CMB001 is directly related to its binding capacity for polystyrene. At 10 μg/mL, pre-treatment with CMB001 resulted in complete inhibition of cell adhesion (Figure 4B), whereas at the same concentration, control cationic peptide (platelet factor 4) had no effect on biofilm formation (not shown). Since CMB001 retains its antibacterial activity when immobilized on a solid surface, it could potentially be used to prevent formation of biofilms on the surface of implants and other medical devices, which often underly post-surgery infections (Arciola et al., 2018).

### Frequency of Resistance to CMB001

The frequency of microbial acquisition of resistance to CMB001 was determined for S. aureus ATCC 29213 and two clinical drug resistant strains, MDR S. aureus CH-11 and A. baumannii (CH-46) (Table 2). The frequency of acquiring resistance for the S. aureus ATCC 29213 strain ranged from 1.8 × 10^–8^ at 2 × MIC of CMB001 to < 5.3 × 10^–10^ at 5 × MIC. No resistant colonies appeared at 10 × MIC. Similar frequencies were calculated for MDR-resistant *S. aureus* CH-11. Under the same conditions, the propensity to develop resistance to nisin was markedly different (Table 2). At 10 × MIC of nisin, the frequency of resistance for *S. aureus* ATCC 29213 was 5.8 × 10^–8^, at least 2-log higher than that of CMB001 at 10 x MIC. The frequency of acquiring resistance to CMB001 for *A. baumannii* CH-46 was 1.2 × 10^–11^ at 4 × MIC, which is 2.5 log lower than to the control antibiotic, colistin. No resistant colonies were detected for CMB001 at 5× and 10× MIC, whereas resistance to colistin was 5.4 × 10^–8^ and 2.0 × 10^–9^ at 5× and 10× MIC, respectively. This initial evaluation of resistance profiles serves for comparative purposes to demonstrate the superiority of CMB001 over nisin. To complete a frequency of resistance profile for CMB001, susceptibility of well-defined strains in liquid cultures will be determined.

**TABLE 2:** Frequency of spontaneous resistance of *S. aureus* (ATCC 29213 and MDR CH-11) and MDR *A. baumannii* against CMB001.

MIC	Strain	Frequency of Resistance to antibiotics			
		
		CMB001	Nisin	Methicillin	Colistin
2X	*S. aureus* (ATCC 29213)	1.3 × 10^–8^	resistant	< 3.72 × 10^–10^	n/d
	MDR *S. aureus* (CH-11)	3.5 × 10^–9^	resistant	resistant	n/d
	*A. baumannii* (CH-46)	1.1 × 10^–7^	n/d	n/d	2.7 × 10^–7^
3X	*S. aureus* (ATCC 29213)	2.2 × 10^–9^	resistant	< 3.72 × 10^–10^	n/d
	MDR *S. aureus* (CH-11)	1.8 × 10^–9^	resistant	resistant	n/d
	*A. baumannii* (CH-46)	2.9 × 10^–9^	n/d	n/d	3.7 × 10^–8^
4X	*S. aureus* (ATCC 29213)	2.6 × 10^–9^	resistant	< 3.72 × 10^–10^	n/d
	MDR *S. aureus* (CH-11)	2.9 × 10^–8^	resistant	resistant	n/d
	*A. baumannii* (CH-46)	1.2 × 10^–11^	n/d	n/d	5.2 × 10^–8^
5X	*S. aureus* (ATCC 29213)	3.7 × 10^–10^	resistant	< 3.72 × 10^–10^	n/d
	MDR *S. aureus* (CH-11)	5.3 × 10^–10^	resistant	resistant	n/d
	*A. baumannii* (CH-46)	< 1 × 10^–11^	n/d	n/d	5.4 × 10^–8^
10X	*S. aureus* (ATCC 29213)	< 3.7 × 10^–10^	5.7 × 10^–8^	< 3.72 × 10^–10^	n/d
	MDR *S. aureus* (CH-11)	< 3.18 × 10^–10^	resistant	resistant	n/d
	*A. baumannii* (CH-46)	< 1 × 10^–11^	n/d	n/d	2.0 × 10^–9^

*The frequency of resistance was calculated as the ratio of colonies growing on antibiotic-containing plates to the total number of colony-forming units in the initial test inoculum (*n* = 3). <denotes that no colonies were observed on test plates (*n* = 3) at a given MIC. n/d, not determined, resistant – colonies grew uninhibited and were too numerous to count.*

### Structural Determination of CMB001

**FIGURE 5:**
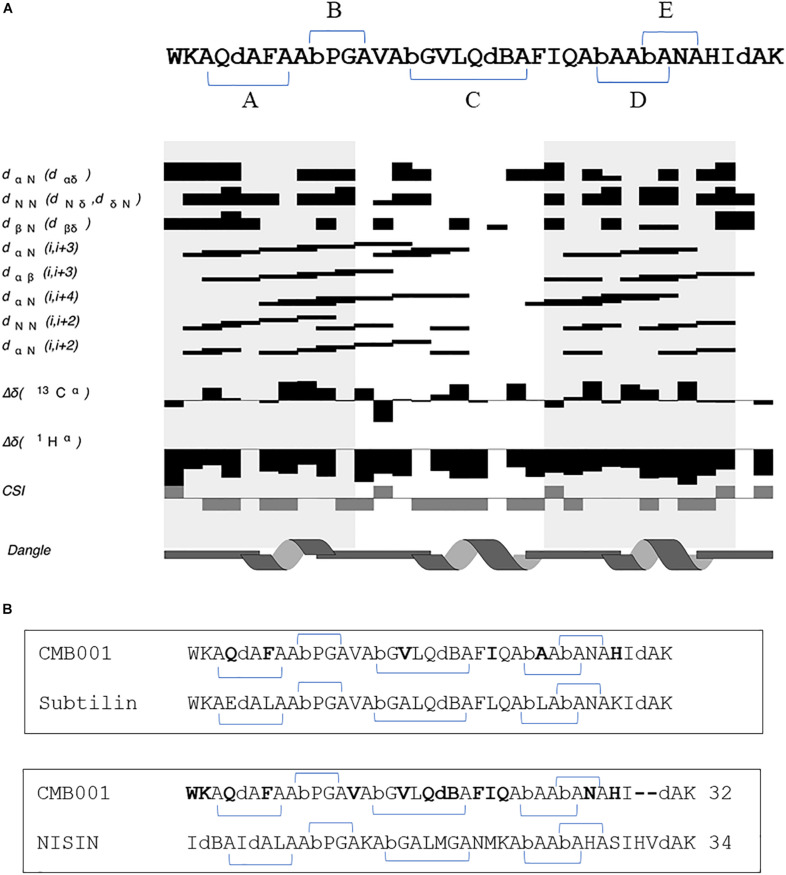
**(A)** Summary of inter-residue Nuclear Overhauser Effects (NOEs) and prediction of secondary structure of CMB001 using 1Hα chemical shifts. The sequence of CMB001 is depicted on top of the graphics with thioether links in brackets and the lanthionine ring topology is labeled by the capital letters **(A–E)**. dA stands for 2,3-didehydroalanine, dB stands for (Z)-2,3-didehydobutyrine, and Ab stands for α-aminobutyric acid. Legend labels are as follows: dαN indicates residues with Hα (i) to HN (i + 1) NOE connections, dNN indicates residues with HN (i) to HN (i + 1) NOE connections, dβN indicates residues with Hβ (i) to HN (i + 1) NOE connections, dαN (i,i + 3) indicates residues with Hα (i) to HN (i + 3) NOE connections, dαβ (i,i + 3) indicates residues with Hα (i) to Hβ (i + 3) NOE connections, dαN (i,i + 4) indicates residues with Hα (i) to HN (i + 4) NOE connections, dNN indicates residues with HN (i) to HN (i + 2) NOE connections, dαN (i,i + 2) indicates residues with Hα (i) to HN (i + 2) NOE connections, Δδ(1Hα) indicates 1Hα difference in chemical shift to sequence adjusted random coil chemical shift for residue type, Chemical Shift Index (CSI) indicates CSI values for residues where −1 indicates α-helix, and +1 indicates β-sheet. For the (i,i + 1) NOE connections, the thickness of the bar indicates the intensity of the NOE cross peak. At the bottom of the graphics, the secondary structure for the peptide is shown. **(B)** Amino acid sequence alignments between CMB001 and two closest lantibiotics, subtilin and nisin. Amino acids unique for CMB001 are shown in bold text.

The structure of CMB001 was elucidated using a series of NMR experiments. The primary and secondary structures of the CMB001 peptide were obtained using 2D NMR. 98% of all hydrogen atoms were assigned using a combination of 2D NOESY, TOCSY, COSY, 15N HSQC, and 13C HSQC spectra. The sequence derived from *de novo* MS/MS analysis was verified using through-space nuclear Overhauser effects (NOEs) (Table 3). CMB001 was identified as a 32 amino acid peptide containing several non-proteinogenic amino acids: 2,3-didehydroalanine, (Z)-2,3-didehydobutyrine, and α-aminobutyric acid (Figure 5A). Amino acid sequence alignments revealed that CMB001 shares 81% homology with subtilin and 62% with nisin (Figure 5B). Based on its primary structure and the lanthionine ring topology, CMB001 could likely be classified as a class I lantibiotic. Elucidation of the biosynthetic pathway will be necessary to provide an accurate classification of this new lantibiotic.

**TABLE 3:** Quality attributes of the CMB001 ensemble structure.

Summary of conformationally restricting experimental constraints	
NOE-based distance constraints:	
Total	425
intra-residue [i = j]	146
sequential [| i - j | = 1]	−149
medium range [1 < | i - j | < 5]	117
long range [| i − j | ≥ 5]	13
NOE constraints per restrained residue	13
Dihedral-angle constraints:	30
Total structures computed	60
Number of structures used	10
Upper distance violations/structure	
0.1 − 0.2 Å	0.7
0.2 − 0.5 Å	2.0
>0.5 Å	4.4
RMSof distance violation/constraint	0.08144 Å
Maximum distance violation	2.01 Å
RMS of dihedral angle restraint/structure	1.6^*o*^
Maximum dihedral violation	70^*o*^
Structure Quality Factors - overall statistics:	
MolProbity clashscore	12.11
Ramachandran Plot Statistics:	
Most favored regions	83.1%
Allowed regions	15.5%
Disallowed regions	1.4%

**FIGURE 6:**
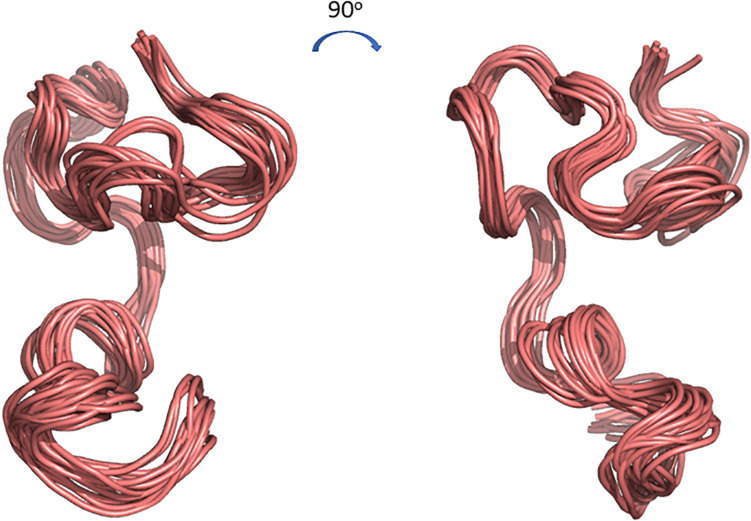
Cartoon representations of the 3D structure ensemble of CMB001. Overlay 15 chosen as a representative ensemble based on distance restraint violations and a converged backbone Root-Mean-Square Deviation (RMSD). Models were created using The PyMOL Molecular Graphics System, Version 2.0 Schrödinger, LLC.

The 3D structure of CMB001 was determined in DMSO using distance and dihedral angle restraints from assigned NOE cross peaks and chemical shifts. Structure calculations were performed using the Gromacs molecular dynamics suite and in-house protocols; NOE distance restraints were iterated until a converged structure ensemble was achieved with a backbone RMSD of 0.52 Å. The final structure ensemble (Figure 6) indicates a compact backbone structure with three pseudo-α-helical regions consisting of residue ranges 8Thr-12Val, 13Thr-19Cys, and Ile21-28Cys.

**FIGURE 7:**
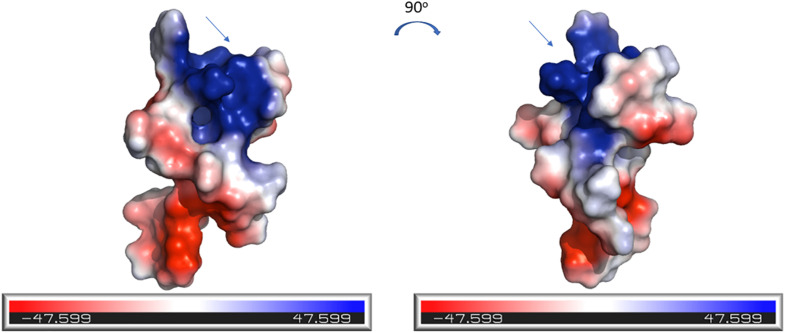
Electrostatics visualization of the surface of CMB001. The blue end of the color gradient represents positive charge and the red end represents negative charge. The arrow indicates the N-terminal region of the molecule. Visualization was generated using the PyMOL Molecular Graphics System, Version 2.0 Schrödinger, LLC.

The electrostatic potential surfaces, calculated using PyMOL software, are shown in Figure 7. The distinct patches of negatively and positively charged regions of the molecule are evenly distributed across its surface. The N-terminal region of CMB001, likely a lipid II binding site, consists of positively charged (blue) and negatively charged (red) patches.

**FIGURE 8:**
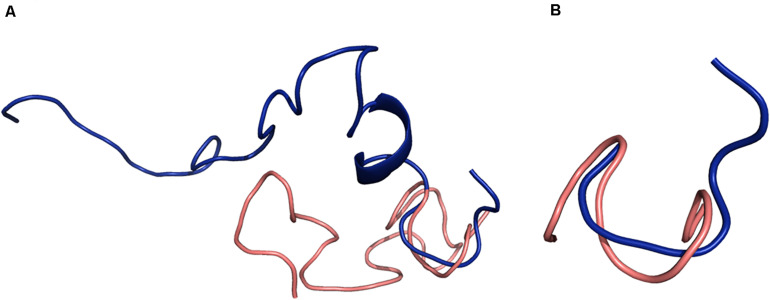
Structural alignments between CMB001 (salmon) and nisin (blue). **(A)** Overall alignment. **(B)** Alignment of the pyrophosphate cage. Blue depicts nisin and salmon depicts CMB001. Alignments were generated using the PyMOL Molecular Graphics System, Version 2.0 Schrödinger, LLC.

A search through the PDB database revealed a partial match for CMB001 with nisin-lipid II (1WCO), but none of the lantibiotics with known structures showed significant structural similarity to CMB001. A structural alignment of CMB001 and nisin was generated using PyMOL(Figure 8). Residues 3-7 were aligned with a pairwise RMSD of 0.5 Å for Calpha atoms, showing that the pyrophosphate region of nisin is structurally similar to that of CMB001 (Figure 8B). A similar alignment was conducted for the residue range 8-12 with a pairwise RMSD of 0.5 Å, showing very good alignment of this structural motif between CMB001 and nisin. A weak alignment was seen for the residue range 12-19 with a pairwise RMSD of 2.8 Å. No structural alignment was possible in the C-terminal region, since nisin lacks defined secondary structure in this region and is highly unstructured there.

## Discussion

Lantibiotics are a class of natural oligopeptides and are a promising area for the discovery of new antibiotics, with several new examples identified in recent years (Sandiford, 2015). These peptides typically have a net positive charge and act through inhibition of cell wall biosynthesis and/or disruption of membrane integrity through the formation of pores (Dischinger et al., 2014). Lantibiotics appear to have similar antimicrobial susceptibility profiles and a common killing mechanism (Dischinger et al., 2014) but differ in their solubility, stability, and frequency of inducing resistant colonies of target pathogens. In the current study, we reported on the discovery, and biological and structural characterization of a novel lantibiotic isolated from a Paenibacillus sp. This peptide, designated CMB001, is active against a wide range of Gram-positive bacteria and select Gram-negative bacteria: *A. baumannii* and *Y. pestis*. While we have described basic fermentation conditions and a purification process, it remains to be established if the production of CMB001 is limited by self-toxicity or could be further improved by optimization of fermentation conditions or adaptation of the producer strain by directed evolution. At laboratory scale, we have been able to produce several 50-100 mg batches of highly purified material to support detailed biological and structural analysis. Since CMB001 shares 81% identity with subtilin and 62% with nisin, it is likely anotherclass I lantibiotic (Figure 5B).

NMR studies of lantibiotics in solution have provided valuable insights into their spatial organization leading to a better understanding of their mode of action. The early study of nisin in aqueous solution at pH 3.5 indicated that two domains, one containing residues 3 – 19 and comprising the first three lanthionine rings, and a second containing the ring system formed by residues 23 – 28, are connected by a flexible hinge region around methionine 21 (van de Ven et al., 1991). NMR analysis revealed that subtilin is also a rather flexible molecule and its overall conformation is similar to that of nisin (Chan et al., 1992). The analysis of the major degradation products of nisin, formed during isolation or storage, revealed that increased flexibility related to the removal of Ala5 and opening of ring A resulted in a loss of antimicrobial activity (Lian et al., 1992). The overall tertiary structure of nisin in the presence of non-aqueous environments is similar to the structure obtained in aqueous solution, with minor conformational changes limited to its N-terminal region (van den Hooven et al., 1993). NMR analysis of the nisin-lipid II complex in DMSO revealed that residues within ring A and B of nisin form a binding site for lipid II (Hsu et al., 2004). The comparison of CMB001 with structures of nisin and subtilin revealed several similarities but also unique regions, which likely define the unique conformational structure of CMB001 and its biological properties. At its N-terminus, CMB001 contains an aromatic residue, tryptophan, followed by a positively charged residue, lysine. The corresponding positions in nisin are occupied an aliphatic isoleucine followed by 3-didehydobutyrine. The replacement of this isoleucine with tryptophan potentiated the antimicrobial activity of nisin, whereas its replacement with charged amino acids had negative effects (Lagedroste et al., 2019). Ring A of CMB001 consists of a e-neutral, polar glutamine, a 2,3-didehydroalanine, and an aromatic phenylalanine, which reduce hydrophobicity relative to nisin. The N-terminal region of CMB001 forms a structure that aligns very well with the corresponding region of nisin (Figure 8). Therefore, despite limited sequence similarity in the N-terminal regions of these peptides, a similar interaction with the pyrophosphate moiety of lipid II is predicted for CMB001 as for nisin.

Rings B and C of nisin are connected by an amphipathic lysine (K12). In CMB001, a hydrophobic residue, valine, is present at this position. It has been shown, that K12 is one of the key residues allowing nisin to adopt a specific conformation within the bacterial membrane (Medeiros-Silva et al., 2018). The presence of a hydrophobic residue at this position in CMB001 could have a significant impact on the interaction of this peptide with the membrane. Rings B and C of CMB001 overlap with subtilin, with the exception of V15 (A15 in subtilin) and therefore, interactions with the membrane could be similar for both molecules.

The hinge region of CMB001, linking rings C and D, is formed by phenylalanine (F20), isoleucine (I21), and glutamine (Q22). By contrast, in nisin, this region is comprised of asparagine, methionine and lysine residues. The flexibility within this region allows nisin to extend along the bacterial membrane and form a pore (Medeiros-Silva et al., 2018). Mutations in the hinge region have been shown to modulate nisin’s antimicrobial activity (Field et al., 2008) and affect its resistance profile (Zaschke-Kriesche et al., 2019b). In CMB001 this hinge region is at the immediate N-terminus of a pseudo-α-helical structure comprising residues I21 to an alanine(A28), and likely forces the molecule to assume a more rigid structure than that of nisin. Such a rigid structure would make it unlikely that upon binding the membrane CMB001 would fully extend along the membrane and adopt the same conformation as nisin (Medeiros-Silva et al., 2018). Compared to subtilin, the hinge region of CMB001 contains a conservative isoleucine to leucine substitution at position 21. Although these are similar amino acids, it has been shown that isoleucine is typically found in β-sheets, while L-leucine is found primarily in α-helices (Brosnan and Brosnan, 2006), likely resulting in distinct flexibility and conformation within the pore between these molecules. Near its C-terminus, CMB001 contains an aromatic and positively charged histidine residue at position 29. In nisin, this position is occupied by a neutral serine. This residue was identified as one of the critical amino acids involved in nisin’s interaction with the membrane of Gram-positive bacteria (Medeiros-Silva et al., 2018). Mutagenesis studies demonstrated that by replacing this serine with a positively charged histidine, antimicrobial activity against *Lactococcus lactis* is reduced (Field et al., 2012). By contrast, in a separate report, mutated versions of nisin with replacements at position 29 showed 2-fold higher activity toward a range of Gram-negative food-associated pathogens (Field et al., 2012). The C-terminal sequence of CMB001 matches well with subtilin, with the exception of a histidine to lysine substitution at position 29. The presence of an imidazole in the side chain and the weaker positive charge of histidine 29 in CMB001 as compared to an amine-containing side chain and stronger positive charge of lysine 29 in subtilin could likely reduce the interaction of CMB001 with the bacterial membrane.

Aligning residues of 3-12 of CMB001 and nisin results in a pairwise RMSD of 2.9, which indicates that while the individual structural motifs of ring A (residues 3-7) and ring B (residues 8-12) show good alignment between CMB001 and nisin, the overall backbone conformation is quite different. Given that 1WCO is a structure of the complex between nisin and lipid II, while CMB001 is an apo-structure, this indicates that CMB001 undergoes a conformational change in order to bind lipid II, where rings A and B combine to bind the pyrophosphate group of lipid II. Molecular dynamic simulations indicate that rings A and B have relatively fixed backbone conformations when not bound to lipid II, however they vary in orientation with respect to one another as indicated by the simulations (Panina et al., 2020). Solid state NMR data show that the nisin rings A and B have small 15N chemical shift changes when bound to different micelles, further indicating rings A and B have only small conformational differences in DMSO and micelles (Medeiros-Silva et al., 2018). There are no NMR structures published of subtilin, however NMR assignments as well as structure calculations have been performed for subtilin at pH 2.5 (Chan et al., 1992). The pattern of NOE connectivities is similar to that for CMB001 and the structural calculations indicate that the only structural elements that have a well-defined conformation are the lanthionine rings. Compared to nisin and subtilin, CMB001 seems to have a relatively well-defined 3D structure and it is possible that this is due to DMSO solvent effects and the high concentration of CMB001. In this work, the oligomeric properties of CMB001 have not been investigated, however NMR spectra show well defined peaks, indicating CMB001 is monomeric in solution. It should be noted that the structure of CMB001 was elucidated in the presence of DMSO and therefore is likely to change upon binding to its natural ligands. Based on structural alignments, CMB001 adopts a similar conformation for the N-terminal rings as nisin (Figure 8). CMB001 stimulates depolarization of bacterial membrane to a comparable degree as nisin (Figure 3B) and both molecules likely attach to the bacterial membrane through a similar mechanism. It is not clear if due to its more compact structure and smaller size, CMB001 could adapt a conformation within cellular membranes similar to nisin (Medeiros-Silva et al., 2018). To reveal conformational transition upon ligand binding, additional NMR analysis of CMB001-lipid II complexes or computational modeling will be necessary (Seeliger and de Groot, 2010).

We have shown that relative to nisin, CMB001 exhibits a much lower propensity to select resistance for *S. aureus* strains, including a MDR strain. It has been reported that that a two-component system, BraRS, is responsible for *S. aureus* resistance to nisin (Kawada-Matsuo et al., 2013). Activation of the BraSR system by nisin leads to phosphorylation of BraR which triggers expression of an ABC transporter responsible for conferring resistance to nisin A (Hiron et al., 2011; Kawada-Matsuo et al., 2013, 2020). It is likely that due to a distinct structure and distribution of charges, activation of the BraRS system by CMB001 is limited and thus it does not induce resistance.

We have shown that CMB001, at 4 × MIC fully eradicated biofilms pre-formed by either ATCC 29213 or MDR strains of *S. aureus* (Figure 4). A recent study revealed, that at similar concentrations (4-8 × MIC), nisin could only partially inhibit formation of biofilms of multi-drug resistant *S. aureus* isolates from human milk, and was ineffective against pre-formed biofilms (Angelopoulou et al., 2020). When coated on polystyrene, CMB001 retained its killing activity for at least 24 h (Figure 4B). If killing activity is also retained after coating metals and other materials utilized in production of medical devices, CMB001 could be applied to prevent biofilm formation on such devices. Some lantibiotics, including nisin, are moderately active against select Gram-negative bacteria (Thomas et al., 2019), but the mechanism of action for this activity is not fully understood. We demonstrate here that CMB001 is active against drug-resistant *A. baumannii* CH-46 (Table 1) as well as a panel of clinical strains representative for *Acinetobacter sp.* (Supplementary Table 1) and its antimicrobial activity and the resistance profile are comparable or superior to colistin, one of the most widely used therapeutic treatment options for infection with carbapenem-resistant *A. baumannii* (Karakonstantis and Saridakis, 2020). In Gram-negative bacteria, the outer membrane prevents nisin from reaching its target, but its potency is enhanced when the outer membrane is destabilized or permeabilized by metal chelators (Boziaris and Adams, 1999). Since increasing permeability of the outer membrane by PMBN did not potentiate activity of CMB001 against *A. baumannii*, it is unlikely that the action of CMB001 toward Gram-negative bacteria is constrained by the outer membrane.

CMB001 remains highly soluble after overnight incubation at pH 7.0 (Figure 2), while the solubility of nisin under neutral pH is limited and reduced further in the presence of a higher concentration of NaCl (Rollema et al., 1995). In addition, nisin tends to lose its activity as a result of oxidation of methionine residues at positions 17 and 21 (Rollema et al., 1996). In nisin Q (Yoneyama et al., 2008) M21 is replaced by leucine and oxidation of the molecule is reduced by ∼50% relative to nisin, although it can still occur at residue 17. In CMB001, the corresponding amino acids are glutamine and isoleucine, respectively. Therefore, the molecule is less susceptible to oxidation and related loss of activity than is nisin. In subtilin, a significant loss of activity was attributed to chemical modification of dA5 by adjacent E4 (Liu and Hansen, 1992). In CMB001, the fourth residue is glutamine, which likely contributes to increased stability of CMB001 relative to subtilin.

These favorable characteristics of CMB001 in comparison to nisin warrant its further evaluation in animal models of bacterial infection. Efficacy of CMB001 to treat MRSA, *M. tuberculosis* and *A. baumannii* will be of great interest. The detailed knowledge of molecular interactions between CMB001 and its cellular target(s) could accelerate its development as a novel drug candidate along with that of biosynthetic analogs.

## Data Availability Statements

The original contributions presented in the study are publicly available. This data can be found in the Protein Data Bank (PDB) under accession number 7K1Q, and in the Biological Magnetic Resonance Data Bank (BMRB) under accession number 30793.

## Author Contributions

JK and SJS designed the study, elucidated amino acid sequence/structure of CMB001, and analyzed the data. JK and SJS wrote the manuscript. CD provided NMR analysis and modeling. PA-O provided MS/MS sequence analysis. SK designed purification methods, prepared materials for NMR and *in vitro* experiments, and conducted analytical evaluations by HPLC and LC/MS. CB designed and conducted *in vitro* susceptibility experiments. YM designed and optimized fermentation conditions, directed microbiological evaluations. AF conducted fermentations, frequency of resistance and cytotoxicity studies. SS contributed to conception of studies and review of data and revised the manuscript. All authors reviewed the final manuscript.

## Conflict of Interest

The authors declare that the research was conducted in the absence of any commercial or financial relationships that could be construed as a potential conflict of interest.
