# Supplementary material for: Isolation, Characterization and Structure Elucidation of a Novel Lantibiotic From Paenibacillus sp
Source: Front Microbiol. 2020 Nov 24;11:598789. doi: 10.3389/fmicb.2020.598789 (PMC7721686; doi:10.3389/fmicb.2020.598789)
Supplement: Supplementary file 1 [file Table_1.docx]

**Table S1. Minimum inhibitory concentration of CMB001 against clinical strains representative for the *Acinetobacter* genus (group).**

| **Hospital ID** | **Drug Resistance** | **16S ID** | **CMB001**  MIC (µg/mL) | **Imipenem** MIC (µg/mL) | **Colistin**  MIC (µg/mL) |
| --- | --- | --- | --- | --- | --- |
| CH36 | N | *Acinetobacter baumannii* | 10 | 250 | 3.91 |
| CH37 | N | *Acinetobacter calcoaceticus* | 10 | 0.5 | 3.91 |
| CH38 | N | *Acinetobacter baumannii* | 40 | 125 | 3.91 |
| CH39 | N | *Acinetobacter baumannii* | 40 | 15.63 | 3.91 |
| CH40 | N | *Acinetobacter baumannii* | 40 | 31.25 | 5.86 |
| CH41 | N | *Acinetobacter ursingii* | 10 | 1 | 1.95 |
| CH42 | N | *Acinetobacter guillouiae* | 20 | 7.81 | 3.91 |
| CH43 | N | *Acinetobacter modestus* | 5 | 0.5 | 7.81 |
| CH44 | N | *Acinetobacter septicus* | 5 | 1.46 | 1.95 |
| CH45 | Y | *Acinetobacter baumannii* | 20 | 125 | 3.91 |
| CH46 | Y | *Acinetobacter baumannii* | 10 | 250 | 3.91 |
| CH47 | Y | *Acinetobacter baumannii* | 20 | 3.91 | 3.91 |
| CH48 | Y | *Acinetobacter baumannii* | 20 | 7.81 | 3.91 |
| CH49 | Y | *Acinetobacter baumannii* | 40 | 62.5 | 3.91 |
| CH50 | Y | *Acinetobacter baumannii* | 20 | 62.5 | 5.86 |
| CH51 | Y | *Acinetobacter baumannii* | 30 | 31.25 | 3.91 |
| CH52 | Y | *Acinetobacter baumannii* | 20 | 31.25 | 3.91 |
| CH53 | Y | *Acinetobacter baumannii* | 20 | 31.25 | 3.91 |
| CH54 | Y | *Acinetobacter baumannii* | 20 | 7.81 | 5.86 |
| CH55 | Y | *Acinetobacter baumannii* | 10 | 46.88 | 7.81 |
